# Nanostructured Molybdenum-Oxide Anodes for Lithium-Ion Batteries: An Outstanding Increase in Capacity

**DOI:** 10.3390/nano12010013

**Published:** 2021-12-21

**Authors:** Hua Wang, Tianyi Li, Ahmed M. Hashem, Ashraf E. Abdel-Ghany, Rasha S. El-Tawil, Hanaa M. Abuzeid, Amanda Coughlin, Kai Chang, Shixiong Zhang, Hazim El-Mounayri, Andres Tovar, Likun Zhu, Christian M. Julien

**Affiliations:** 1 Department of Mechanical and Energy Engineering, Indiana University-Purdue University Indianapolis, Indianapolis, IN 46202, USA; wanghua@iu.edu (H.W.); tl41@iupui.edu (T.L.); kc59@iu.edu (K.C.); helmouna@iupui.edu (H.E.-M.); tovara@iupui.edu (A.T.); 2 National Research Centre, Inorganic Chemistry Department, Behoes Street, Dokki, Giza 12622, Egypt; achraf_28@yahoo.com (A.E.A.-G.); r2samir@yahoo.com (R.S.E.-T.); hanaa20619@hotmail.com (H.M.A.); 3 Department of Physics, Indiana University, Bloomington, IN 47405, USA; amacough@iu.edu (A.C.); sxzhang@indiana.edu (S.Z.); 4 Institut de Minéralogie, de Physique des Matériaux et Cosmologie (IMPMC), Sorbonne Université, UMR-CNRS 7590, 4 Place Jussieu, 75752 Paris, France

**Keywords:** molybdenum oxides, green synthesis, biological chelator, additional capacity, anodes, lithium-ion batteries

## Abstract

This work aimed at synthesizing MoO_3_ and MoO_2_ by a facile and cost-effective method using extract of orange peel as a biological chelating and reducing agent for ammonium molybdate. Calcination of the precursor in air at 450 °C yielded the stochiometric MoO_3_ phase, while calcination in vacuum produced the reduced form MoO_2_ as evidenced by X-ray powder diffraction, Raman scattering spectroscopy, and X-ray photoelectron spectroscopy results. Scanning and transmission electron microscopy images showed different morphologies and sizes of MoO_x_ particles. MoO_3_ formed platelet particles that were larger than those observed for MoO_2_. MoO_3_ showed stable thermal behavior until approximately 800 °C, whereas MoO_2_ showed weight gain at approximately 400 °C due to the fact of re-oxidation and oxygen uptake and, hence, conversion to stoichiometric MoO_3_. Electrochemically, traditional performance was observed for MoO_3_, which exhibited a high initial capacity with steady and continuous capacity fading upon cycling. On the contrary, MoO_2_ showed completely different electrochemical behavior with less initial capacity but an outstanding increase in capacity upon cycling, which reached 1600 mAh g^−1^ after 800 cycles. This outstanding electrochemical performance of MoO_2_ may be attributed to its higher surface area and better electrical conductivity as observed in surface area and impedance investigations.

## 1. Introduction

Understanding and realization of the benefit of efficient energy storage is one of the most important strategies for achieving sustainable development [1,2]. Nowadays, lithium-ion batteries (LIBs) have become one of the most important energy storage technologies due to the fact of their higher storage capacity and power density compared to other rechargeable batteries [3,4,5]. The development and rapid increase in portable electronic devices and electric vehicles have accelerated the pursuit of developing LIBs with high energy and power densities [6,7]. Therefore, it is essential to develop high-capacity electrode materials for LIBs [8,9,10,11,12,13]. Graphite has become the standard anode material for LIBs since their commercialization by Sony Corporation [14]. However, graphite has relatively low theoretical capacity (372 mAh g^−1^ and 850 mAh cm^−3^), which cannot meet the demand of current large-scale energy applications [15]. To address this issue, there is a continuous effort to explore alternative anode materials. For instance, transition metal oxides (TMOs), such as NiO, MnO_2_, TiO_2_, Fe_3_O_4_, MoO_3_, and MoO_2_, have been studied as anode materials for LIBs. These oxides are abundant, low cost, and have a high theoretical specific capacity of approximately 500–1200 mAh g^−1^ due to the fact of their conversion reaction upon lithiation [16,17,18,19,20,21].

Molybdenum oxides with different oxidation states (e.g., MoO_3_, MoO_3-δ_, Mo_n_O_3n–1_, and MoO_2_) and a broad spectrum of electrical properties ranging from wide band gap semiconducting (MoO_3_) to metallic (MoO_2_) character are considered as promising anode materials for LIBs [22]. Their specific capacities are significantly higher than that of graphite [23,24,25,26,27,28,29]. In particular, MoO_3_ with an orthorhombic crystal structure is a thermal stable, abundant, cost effective, and a rather safe oxide with a theoretical capacity of 1117 mAh g^−1^ and a typical discharge potential plateau around 0.45 V [30,31,32,33,34]. It has a unique layered structure that is convenient for fast lithium diffusion transport [15,35,36,37,38]. The overall first lithiation reaction for MoO_3_ is described by two reactions: the lithium insertion (addition) at a potential >1.5 V up to *x* ≈ 1.2 (Equation (1)) and the conversion (transformation) reaction at a potential <0.5 V up to *x* ≈ 6.0 (Equation (2)) as follows [22]:

MoO_3_ + *x*Li^+^ + *x*e^−^ → Li_x_MoO_3_, (1)

Li_x_MoO_3_ + (6−*x*)Li^+^ + (6−*x*)e^−^→ Mo + 3Li_2_O. (2)

Some drawbacks have been reported for MoO_3_, such as phase transformation accompanied by volume expansion with repeating cycling, which leads to a rapid capacity fading [39,40].

On the other hand, MoO_2_ crystallizes in the monoclinic structure with space group *P*2_1_/*c*, which can be viewed as a distorted rutile phase. This structure is composed of MoO_6_ octahedra joined by edge-sharing, which form a (1 × 1)-tunneling network [41,42]. In addition, MoO_2_ has outstanding properties for energy storage applications, e.g., metal-like conductivity (~6 × 10^3^ S cm^−1^), very low toxicity, cost-effectiveness, high chemical and thermal stability, high volumetric capacity due to the fact of its high density (6.5 g cm^−3^), and high theoretical capacity (838 mAh g^−1^) [43,44,45]. The first lithiation mechanism is an insertion-type reaction that takes place in the bulk and amorphous MoO_2_ electrodes with only one-electron reduction as described by Equation (3) [46]:

MoO_2_ + *x*Li^+^ + *x*e^−^ ↔ Li_x_MoO_2_, (3)

with 0 ≤ *x* ≤ 0.98. The second mechanism is a conversion reaction that gradually resolves Li_x_MoO_2_ as described by Equation (4) [47]:

Li_x_MoO_2_ + (4-*x*)Li ↔ 2Li_2_O + Mo. (4)

that shows the formation of metallic Mo and Li_2_O.

MoO_3_ and MoO_2_ have been prepared in different morphologies, e.g., nanoparticles [48], nanowires [49,50], nanorods [51,52], nanotubes [53], nanosheets [54], and nanobelts [55]. These nanosized fabrications were expected to improve the electrochemical performance [56]. However, these fabrication methods are complicated, expensive, energy- and time-consuming, and non-scalable [57]. To alleviate these limitations, to some extent, an attempt was made to use a rather benign approach via simple, green, and eco-friendly reducing agents for nanoparticles formation [58]. Green synthesized particles have low toxicity and are more stable than those prepared by traditional methods, as biological sources provide a stabilizing and capping effect for the synthesized particles, especially extracts of plants [58].

Extracts of waste products have been used as cost-effective, eco-friendly, and efficient raw materials for various energy storage applications [59,60]. In our previous work, we used extracts of lemon and orange peels to synthesize manganese dioxides, which has been used as cathode materials in LIBs and supercapacitors [60,61]. Further processing and using large quantities of orange peels as a byproduct will reduce hazardous impacts and serious environmental pollution [60]. Orange peels contain polyphenolic and flavonoid compounds which have hesperidin, narirutin, naringin, and eriocitrin [62]. It is well known that the phenolic compounds have at least one aromatic ring. The latter is attached to one or more hydroxyl groups. The number and position of the carboxylic group has a direct impact on reducing the antioxidant ability of flavonoids and phenolic acids. As the number of hydroxyl group increases, the antioxidant activity increases [63]. Orange is considered as one of the most important fruits with a global production of 48.8 (2016/17) million tons. Industrial extraction of citrus juice consumed a large portion of this production. As a result of this industry, there are large amounts of residues, e.g., peel and segment membranes. A high percent of these residues is related to peel byproduct that represents between 50% and 65% of the total weight of the fruit; reported chemical analysis for orange peel showed 7.1% protein and 12.79% crude fiber. In addition, limonoids and flavonoids with antioxidant activity were also found in orange peel. This antioxidant activity of citrus peel extracts comes from glycosides hesperidin and naringin present in this extract. Orange peel also contains coniferin and phlorin as additional phenols that help in radical scavenging when administered in the form of orange peel molasses, and this will promote sustainable disposal of orange peels [64].

In this study, orange peel extract was used as an effective chelating agent to synthesize molybdenum oxides. MoO_3_ and MoO_2_ were prepared by altering the calcination conditions: in air for MoO_3_ and in vacuum for MoO_2_ at a low temperature of 450 °C. The as-prepared oxides were subjected to various characterizations, including X-ray diffraction (XRD), thermogravimetric analysis (TGA), scanning electron microscopy (SEM), transmission electron microscopy (TEM), Raman scattering (RS) spectroscopy, and X-ray photoelectron spectroscopy (XPS), to elucidate their morphological and structural properties. Further electrical and electrochemical characterizations, including cyclic voltammetry (CV), galvanostatic charge–discharge (GCD), electrochemical impedance spectroscopy (EIS), and area-specific impedance (ASI), were carried out for the as-prepared molybdenum oxides as anode materials for LIBs.

## 2. Materials and Methods

Ammonium molybdate, conductive carbon black super C65 (Timcal Co., Bodio, Switzerland), binder polyvinylidene fluoride (PVDF, 12 wt.%, Kureha Battery Materials Japan Co., Tokyo, Japan), solvent 1-methyl-2-pyrrolidinone (NMP, anhydrous 99.5%, Sigma–Aldrich, Burlington, MA, USA), electrolyte 1 mol L^−1^ LiPF_6_ in ethylene and dimethyl carbonate solution mixed as a 1:1 volume ratio (BASF Corporation, Ludwigshafen-am-Rhein, Germany), and lithium ribbon (thickness 0.38 mm, 99.9% trace metals basis, Sigma–Aldrich, Burlington, MA, USA) were employed as received.

**Figure 1:**
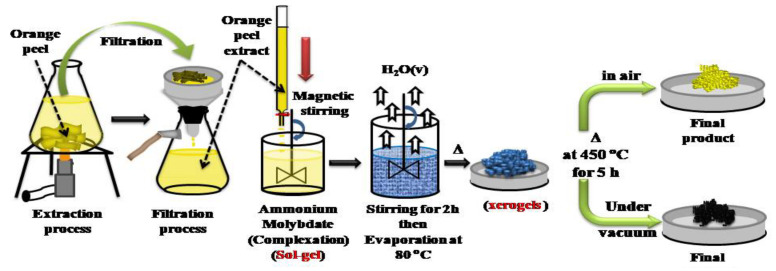
Schematic diagram for the synthesis of MoO_3_ (yellow color) and MoO_2_ (black).

Molybdenum oxides were prepared by the sol-gel method using ammonium molybdate tetrahydrate, (NH_4_)_6_Mo_7_O_24_∙4H_2_O as the source of molybdenum, and extract of orange peel as the chelating agent. Pure filtrated extract of orange peel was obtained through boiling small pieces of cleaned waste peels in distilled water at 100 °C for 10 min. A schematic representation of the MoO_3_ and MoO_2_ growth process is shown in Figure 1. Pure orange peel extract drops were added with vigorous stirring to a 100 mL solution of 4 g of (NH_4_)_6_Mo_7_O_24_∙4H_2_O. During this operation, the solution changed in color from yellow to blue until conversion to a dark gel. The dry xerogel (precursor) was divided into two parts: one part was calcined in air at 450 °C for 5 h (MOA, yellow color) and the second was calcined under vacuum at 450 °C for 5 h (MOV, black color).

XRD analyses of as-prepared samples were processed using a Bruker D8 Discover XRD Instrument equipped with CuK_α_ radiation (λ = 1.5406 Å). The scanning rate was 1.2° min^−1^, for 2*θ* between 10° and 80°. Raman spectra were recorded at room temperature with a micro-Raman spectrometer (Renishaw, Wotton-under-Edge, UK) with a confocal Raman microscope inVia^TM^ system at a 532 nm laser-line excitation. The spectra were calibrated with the reference Si phonon peak at 520 cm^−1^. The morphology of the materials was studied by field emission scanning electron microscopy (FESEM, JEOL JSM-7800F) and by transmission electron microscopy (TEM, JEOL, JEM-2100 microscope, Japan). BET surface area and pore size distribution of synthesized samples were determined from an N_2_-physisorption analyzer (ASAP 2020 system, Micromeritics Corporate, Norcross, GA, USA). The BET surface area was calculated from the isotherms in the range from 0.02 to 0.4 of relative pressures (*P*/*P*_0_). TGA measurements were carried out for the prepared samples using a thermal gravimetric analyzer (Perkin Elmer, TGA 7 series) in a temperature range of 50–1000 °C at a heating rate of 10 °C min^−1^ in air. X-ray photoelectron spectra were recorded using a PHI VersaProbe II Scanning X-Ray Microprobe system equipped with a Mg Kα source (λ = 1253.6 eV).

LIB electrodes were fabricated as a mixture of active materials (Mo oxide powders), carbon black (CB), and polyvinylidene fluoride binder (PVDF) in a 5:3:2 mass ratio. We used a high percentage of carbon black and PVDF binder in the electrode to maintain the mechanical integrity and good electrical connection in the electrode during long-term cycling experiment. The mixture was added to N-methyl-2-pyrrolidone (NMP) solvent. The mixed slurry was magnetically stirred for 24 h to form a homogeneous blend. The well-blended slurry was cast on a copper foil by a doctor blade and was dried under vacuum at 100 °C for 24 h. Finally, electrodes were punched out as ~0.97 cm^2^ discs (Φ = 11 mm). CR2032 coin cells processed in an argon-filled glovebox using 30 µL electrolyte dripped on the electrode, then on a Celgard 2400 separator. Electrochemical tests were carried out using an Arbin BT2000 battery cycler at room temperature. Before cycling, cells were initially maintained at rest for 30 min. Cells were cycled galvanostatically at C/10 and 1C-rate (1C = 838 mA g^−1^ for MoO_2_ and 1C = 1117 mA g^−1^ for MoO_3_) in a voltage range between 0.01 and 3.0 V. Cyclic voltammetry was conducted at room temperature on a BioLogic VSP workstation in which the potential was set to sweep from open-circuit voltage to 0.01 V and then to sweep back to 3.0 V at a 0.02 mV s^−1^ scanning rate. Electrochemical impedance spectroscopy was also conducted by the VSP workstation in the frequency range from 5 × 10^5^ to 0.1 Hz with an amplitude of 5 mV.

## 3. Results

### 3.1. Structure and Morphology

**Figure 2:**
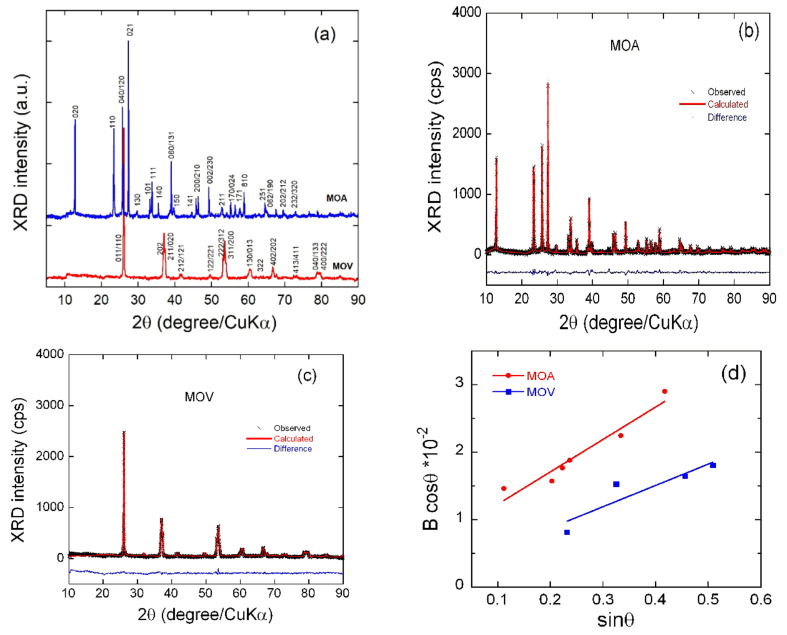
(**a**) XRD patterns of the as-prepared MOA and MOV samples. (**b**) Rietveld refinement of the MOA sample. (**c**) Rietveld refinement of the MOV sample. Cross marks are experimental data and solid lines (in red) are calculated diagrams. The curve at the bottom is the difference between the calculated and observed intensities. (**d**) Analysis of micro-strain from the full-width *B* at half-maximum of the XRD peaks according to Equation (1).

The X-ray diffractograms of MOA and MOV materials are shown in Figure 2a. Patterns display well-resolved reflections with a very smooth background indicating the high crystallinity of Mo oxides prepared by the sol-gel method with biological chelating agent and final calcination at 450 °C. The XRD spectrum of MOA exhibited the typical pattern of the α-MoO_3_ phase and can be indexed in the orthorhombic structure with *Pbnm* space group (JPCDS card 76-1003) [65]. The presence of a preferred orientation of (0*k*0) planes was evidenced by the (020), (040), and (060) Bragg lines with large intensities. The XRD spectrum of the MOV material displayed sharp diffraction peaks indicating the formation of highly crystallized MoO_2_, which can be indexed using the monoclinic structure with the *P*2_1_/*c* space group (JPCDS card 68-0135). In order to characterize the phase purity as well as the phase composition, the full structural identification of the MoO_3_ and the MoO_2_ powders were analyzed using Rietveld refinements. The results are listed in Table 1, and the refined XRD spectra are displayed in Figure 2b,c. The small values of the residual and reliability parameters (*R*_p_, *R*_w_, and *χ*^2^) of the Rietveld refinement indicate the successful identification of the orthorhombic and monoclinic phases of MoO_3_ and MoO_2_ powders, respectively, even in the presence of some impurity phases as in the case of MOV. The lattice parameters obtained from Rietveld refinement are in good agreement with values of our previous work as well as other literature [22,66,67,68]. A careful examination of the MoO_2_ sample calcinated in vacuum reveals the presence of a small amount of Mo-suboxides such as Mo_4_O_11_, Mo_8_O_23_, and Mo_9_O_26_ (Table 1). These compositions belong to Mo_n_O_3n–1_ suboxides (Magnéli phases, *n* = 4–9), which crystallize into the ReO_3_-type structure characterized by the presence of empty channels due to the loss of oxygen [23,68,69]. These compositions (Mo_4_O_11_, Mo_8_O_23_, and Mo_9_O_26_) deduced form Rietveld refinement imply the presence of a mixture of Mo with oxidation states between +6 and +4. This electronic configuration implies a concentration of free carriers and, thus, a large electrical conductivity, which is beneficial to electrochemical properties [68].

The formation of Mo_4_O_11_, Mo_8_O_23_, and Mo_9_O_26_ suboxides under vacuum is due to the presence of not only the ammonia in ammonium molybdate but also the CO and CO_2_ gases generated by the combustion reaction of carbon found in the organic components of orange peel (i.e., ascorbic and citric acid) which reduce the Mo^6^^+^ ions in absence of O_2_. The average crystalline sizes of the prepared samples were calculated using the Debye–Scherrer’s formula from the full-width of diffraction peaks. They were found to be ≈29 and 45 nm for MOA and MOV, respectively. Further information on the structural properties can be obtained from the broadening of diffraction peaks that is considered an indicator, not only of the crystallinity of the MOA and MOV powder, but also of the homogeneous distribution of cations over the structure. The micro-strain (ε) of the MOA and MOV particles was determined using the Williamson–Hall equation [70]:

*B*_hkl_ cos *θ*_hkl_ = (*K*λ/*L*_c_) + 4*ε* sin *θ*_hkl_ (5)

where *B*_hkl_ is the line broadening of a Bragg reflection (*hkl*), *K* is the shape factor, *L*_c_ is the effective crystallite size, and λ is the X-ray wavelength. The micro-strain is estimated from the slope of the plot (*B*_hkl_ cos *θ*_hkl_) vs. (sin *θ*_hkl_) and the intersection with the vertical axis provides the crystallite size. The *B*_hkl_ value used here was the instrumentally corrected one. From Figure 2d, the micro-strain was determined to be 11.9 × 10^−^^2^ and 7.9 × 10^−^^2^ rd for MOA and MOV, respectively, showing a slight difference in the crystallinity of the samples, as the micro-strain was strongly affected by the heat-treatment conditions.

**Figure 3:**
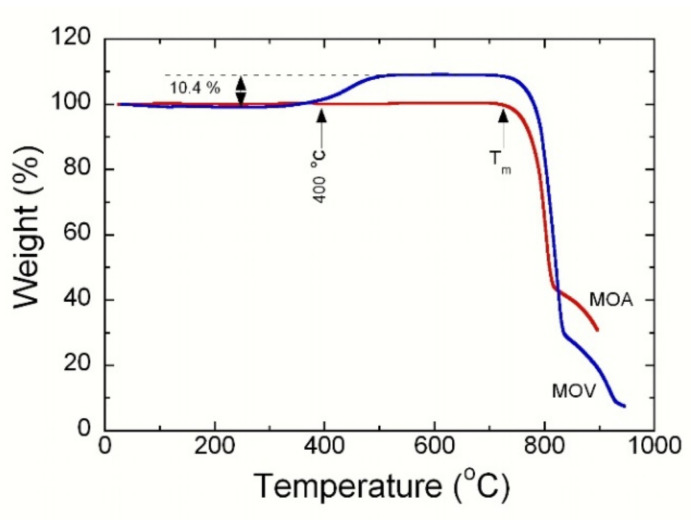
Thermogravimetry (TG) curves of the MOA and MOV samples recorded at a heating rate of 10 °C min^−1^. MOA displays the typical behavior of the stoichiometric MoO_3_ orthorhombic phase, while MOV shows the oxidation of the suboxide at 400 °C and the conversion to MoO_3_ at 600 °C.

Figure 3 displays the thermogravimetry (TG) curves of MOA and MOV samples recorded at a heating rate of 10 °C min^−1^. The MOA sample showed a stable, flat, and straight-line TG profile without weight change until the start of decomposition above 730 °C. These features indicate a stochiometric MoO_3_ material without any oxygen vacancies or carbon coating due to the burning of the extract of organic peel. On the contrary, the thermal behavior of reduced MOV looked different. The TG curve was stable and flat until approximately 400 °C. Above 400 °C, a gradual and pronounced weight gain occurred until reaching the highest value of 10.4% weight gain, which was due to the re-oxidation and filling of oxygen vacancies in the suboxide (Mo_4_O_11_, Mo_8_O_23_, and Mo_9_O_26_) lattices, and the transformation of MoO_2_ to the stoichiometric MoO_3_ phase at *T* = 600 °C. Theoretically, the weight gain for the conversion of MoO_2_ to MoO_3_ was approximately 12.5%. From the TG analysis, it was observed that the weight of the sample increased by ≈10.4% from room temperature to 600 °C in the air. Thus, the calculated value for conversion of MoO_2_ and the suboxides (i.e., Mo_4_O_11_, Mo_8_O_23_< and Mo_9_O_26_) to the stoichiometric MoO_3_ is close to the theoretical value. It is worth noting that the color of MoO_2_ was black before the TG measurements and converted to the color yellow after the TG runaway to 600 °C. In addition, this TG of MOV confirmed that there was no carbon coating around their particles. This was because there was no weight loss above 500 °C related to the emission of CO_2_ as a result of the reaction between oxygen in the air and carbon, if present. However, some mass loss might occur, which was masked by oxygen gain during the oxidation process.

**Figure 4:**
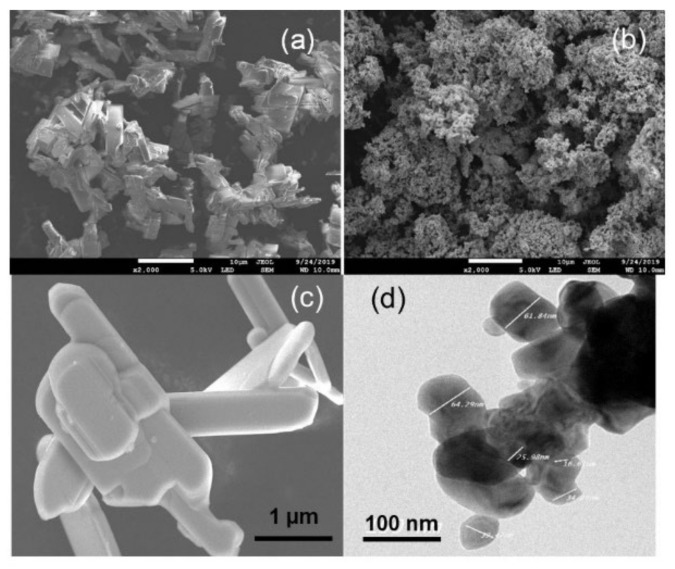
(**a**,**b**) SEM images of the MOA and MOV samples synthesized using a sol-gel method assisted by a biological chelator (scale bar of 10 µm). (**c**) Magnified SEM image of the MOA (scale bar of 1 µm) and a (**d**) TEM image of the MOV sample (scale bar of 100 nm).

The SEM images (a–c) and TEM image (d) of the MOA and MOV samples depicted in Figure 4 illustrate the influence of the synthesis conditions on the particle size and morphology. There is a significant difference between the morphology of molybdenum oxide prepared in air and that prepared in vacuum from the same ammonium molybdate tetrahydrate precursor. Air calcination gives heterogeneous MOA particles with well-crystallized crystals a platelet-like shape (Figure 4a,c). The sizes of the MOA particles were in a wide range from the sub-micrometer to ~10 µm [71]. On the contrary, calcination in vacuum provided homogeneous MOV powders with an ash-like morphology at the nanometer size (Figure 4b). SEM (Figure 4c) and TEM images (Figure 4d) show that the MOA platelets had sizes larger than 1 µm, while the MOV powders had smaller sizes, in the 40–100 nm range. The size and morphology differences of the MOA and MOV particles were mainly due to the heat treatment conditions.

**Figure 5:**
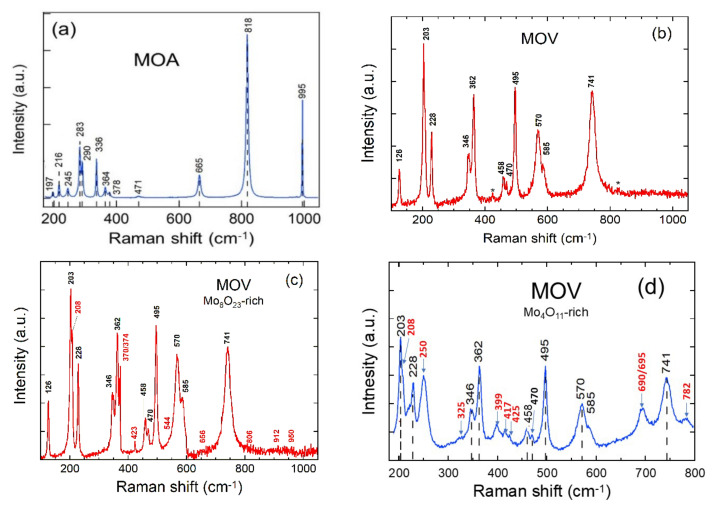
Raman spectra of the (**a**) MOA powders, (**b**) MOV (MoO_2_-rich sample where the stars correspond to Raman peaks of suboxides), (**c**) MOV with m-Mo_8_O_23_-rich particles, and (**d**) MOV with o-Mo_4_O_11_-rich particles. Spectra were recorded at a spectral resolution of 1 cm^−1^.

Raman spectroscopy is a sensitive tool for investigating the coordination, structure, lattice vibrations, and symmetry of molybdenum and oxygen atoms in the presence of different phases. Further structural analyses of as-prepared molybdenum oxides were carried out by Raman scattering spectroscopy using the excitation line at λ_exc_ = 532 nm (Figure 5a–d). The Raman spectrum of the MOA sample (Figure 5a) displays the typical vibrational features of the orthorhombic α-MoO_3_ phase. Twelve vibrational modes were evidenced by the peaks located at 197, 216, 245, 283, 290, 336, 364, 378, 471, 665, 818, and 995 cm^−1^. The wavenumbers and relative intensities matched closely with the single crystal Raman spectrum given in the literature [72,73,74,75,76]. Most of the Raman active modes were dominated by either interlayer or intralayer contributions. More specifically, the peak at 665 cm^−1^ was related to the ν(O-Mo_3_) stretching mode of the triply-coordinated oxygen atoms, which are shared by three MoO_6_ octahedra. The intense peak at 818 cm^−1^ was linked to the doubly-coordinated oxygen ν(O-Mo_2_) stretching mode. The high-wavenumber peak at 995 cm^−1^ was associated with the ν(Mo^6+^ = O) asymmetric stretching mode of terminal singly-coordinated (unshared) oxygen atoms, which had bonds that were responsible for the layered structure of the α-MoO_3_ orthorhombic phase [75]. Figure 5b–d presents the micro-Raman spectra of the MOV sample recorded on different areas of the sample using 1% laser power (0.5 mW) at a 532 nm laser-line excitation. The micro-Raman results were in good agreement with the XRD findings. The mixture of vibrational features of the MoO_2_ and Mo_n_O_3n–1_ suboxide phases (i.e., o-Mo_4_O_11_, m-Mo_8_O_23_, o-Mo_9_O_26_ Magnéli) can be identified [77,78,79,80,81,82,83]. The Raman bands of the MOV sample located at 126, 203, 228, 346, 362, 458, 470, 495, 570, 585, and 741 cm^−1^ (Figure 5b) correspond to a rutile-type (monoclinic) structure and agreed well with the vibrational features of the m-MoO_2_ reported in the literature [77,78,79,80]. Two weaker peaks, located at 425 and 820 cm^−1^, were attributed to the orthorhombic o-Mo_4_O_11_ suboxide [81,83].

Vibrational analysis of the MoO_2_ spectrum reveals that the bands in the 500–800 and 200–400 cm^−1^ regions were due to Mo–O stretching and bending modes, respectively. The low-frequency region (<200 cm^−1^) corresponded to the lattice modes. The Raman spectra of suboxide-rich areas are displayed in Figure 5c,d for the MOV with m-Mo_8_O_23_-rich and o-Mo_4_O_11_-rich particles, respectively (Raman peaks of suboxides are marked in red). The monoclinic m-Mo_8_O_23_ phase is identified through the Raman peaks at 208, 370/374, 656, 912, and 950 cm^−^^1^, whereas peaks located at 208, 250, 325, 399, 417, 425, 695, 782, 806, and 912 cm^−^^1^ are assigned to the o-Mo_4_O_11_ phase. Moreover, the MOV sample contained a small amount of the o-Mo_9_O_26_ suboxide identified by the peaks at 208, 544, 782, 912, and 950 cm^−^^1^. The spectroscopic results are listed in Table 2 and compared with the literature data [81,82].

**Figure 6:**
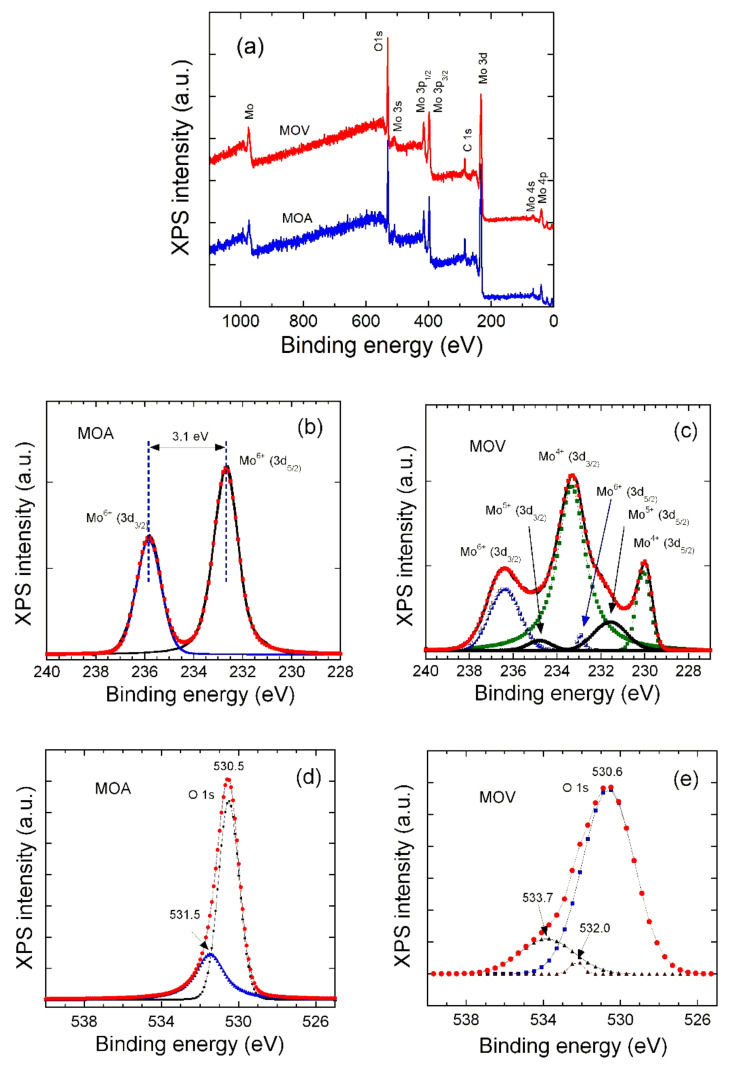
(**a**) XPS survey spectra of the MOA and MOV samples. High-resolution XPS spectra of (**b**) Mo 3d in MOA, (**c**) Mo 3d in MOV, (**d**) O1s in MOA, (**e**) O1s in MOV.

XPS measurements were carried out to evaluate the chemical composition and investigate the surface valance states of Mo in MOA and MOV samples. The results are shown in Figure 6. The survey spectra (Figure 6a) display the fingerprints of the Mo 3d, Mo3p_3/2_, Mo3p_1/2_, and O1s core levels (their binding energies are listed in Table 3). The Mo 3d and O1s peaks were analyzed by evaluating the peak area of elements using Gaussian profiles after removing the secondary electron background. All XPS spectra can be deconvoluted using two Mo 3d doublets with 3d_5/2_ and 3d_3/2_ species. For the MOA sample (Figure 6b), the Mo 3d_5/2_ and Mo3d_3/2_ characteristic peaks were located at 232.6 and 235.7 eV, respectively (with a spin–orbit separation of ~3.1 eV), suggesting the sole existence of Mo^6+^ species on the MOA surface [23,84,85]. The binding energy of the Mo 3d_5/2_ line for polycrystalline MoO_3_ has been reported to be 231.6–s232.7 eV [86,87,88]. For the MOV sample, the deconvoluted peaks in Figure 6c unambiguously reveal the co-existence of mixed Mo valence states (Table 3).

The first doublet centered at 230 and 233.3 eV was typically the Mo 3d_5/2_ and Mo 3d_3/2_ of Mo^4+^, respectively, whereas the second one located at 231.6 and 234.7 eV was due to the Mo^5+^, and, finally, the last one located at 233.1 and 236.3 eV was due to the Mo^6+^ [88,89]. The XPS spectra of O1s are presented in Figure 6d,e for the MOA and MOV samples, respectively. The intense peak located at ∼530.5 ± 0.1 eV was attributed to the binding energy of Mo–O bonds, whereas the peak at a higher binding energy was assigned to surface states. Therefore, the surface states of the MOV observed in the XPS patterns ascribed the presence of MoO_2_, Mo_4_O_11_, Mo_8_O_23_, and Mo_9_O_26_ (Figure 6c). From XPS peak deconvolution, the average Mo valence state of the MOA and MOV sample was determined to be 6.00 and 4.39, respectively.

The porous texture of the MOA and MOV samples was investigated by the N_2_ adsorption–desorption isotherm measurement. The isotherm profiles of samples can be categorized as a type IV curve with a H3 hysteresis loop at the relative pressure of 0.8–1.0, thus implying the existence of a large number of mesopores. The average pore size was below 2 nm in the MOV material, while the MOA sample exhibits an average pore size of 10 nm. Moreover, the Brunauer–Emmett–Teller (BET) specific surface area of the MOV was 4.0 m^2^ g^−1^, which was higher than that of the MOA (0.23 m^2^ g^−1^). The mesoporous structure of the Mo–O samples may be beneficial for the electrolyte to penetrate completely into the pores and diffuse efficiently to active sites with less resistance, and can also buffer large volume change during the Li^+^-ion insertion/extraction processes. The equivalent particle size of the MOA and MOV samples can be calculated from the BET data and compared using SEM images. The average particle size (nm) is expressed by Equation (6) below [90]:

(6) $$L_{\mathrm{BET}}=\frac{6000}{S_{\mathrm{BET}}d},$$

where *S*_BET_ is the specific surface area (in m^2^ g^−1^) measured by BET experiments, and *d* is the gravimetric density (4.70 and 6.47 g cm^−3^ for MoO_3_ and MoO_2_, respectively). Results of the sample texture are summarized in Table 4. Note that the *L*_BET_ values corresponded to the average size of the secondary particles (agglomerates observed in SEM images).

### 3.2. Electrochemical Properties

**Figure 7:**
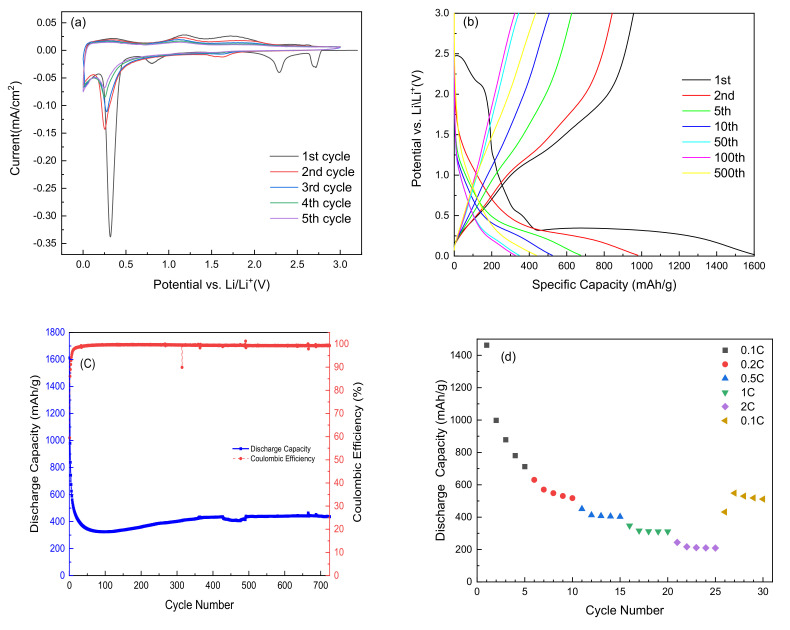
Electrochemical performance of MOA as anode material: (**a**) five first cyclic voltammograms recorded 0.01 mV s^−1^ scanning rate, (**b**) galvanostatic charge/discharge curves carried out at 1C-rate (~1.1 A g^−1^) in the potential window 0.01–3.0 V vs. Li^+^/Li, (**c**) specific discharge capacity and Coulombic efficiency as a function of cycle numbers performed at a 1C-rate, and (**d**) rate capability.

The electrochemical properties of as-prepared MOA and MOV as anode materials of LIBs were investigated in a potential range of 0.01–3.0 V vs. Li^+^/Li. Figure 7a shows the cyclic voltammetry (CV) curves of MoO_3_ performed at a scanning rate of 0.01 mV s^−1^. MoO_3_ demonstrates four prominent peaks in the first discharge process located at 2.7, 2.28, 0.7, and 0.3 V. The peaks at 2.7, 2.28, and 0.7 V appear only in the first discharge cycle and disappear in the subsequent cycles. This feature has been attributed to the intercalation of Li ions into the interlayer space between MoO_6_ slabs, which occurs as the Li_x_MoO_3_ phase (see Equation (1)) and causes irreversible structural change to MoO_3_ in a lithiated amorphous phase [91]. The peak at 0.3 V originates from a conversion reaction (see Equation (2)) of Li_x_MoO_3_ to Mo^0^ and Li_2_O [92]. The shift to a rather low voltage for the peak at 0.3 V with subsequent cycles may be attributed to a structure evolution. Two broad anodic peaks observed at approximately 1.18 and 1.73 V correspond to the de-lithiation process and are maintained in the forthcoming cycles. Note that the strong cathodic peak slightly shifts by 0.25 V after the first cycle. Figure 7b displays the galvanostatic charge–discharge profiles of the MOA sample. The upper voltage discharge plateau, observed in the first discharge, disappear in the subsequent cycles and the plateau at approximately 0.3 V shifts to lower voltage as noticed in CV results. These electrochemical features are those of the MoO_3_ phase reported so far [93]. At 1C-rate (current density of ~1.1 A g^−1^), the discharge capacity of the MoO_3_ electrode decreased abruptly from the initial value of 1613 to 330 mAh g^−1^ over the first 50 cycles and then slightly increased in subsequent discharge-charge cycles at a rate of 0.35 mAh g^−1^ per cycle, reaching the specific capacity of 435 mAh g^−1^ after 725 cycles (Figure 7c). The Coulombic efficiency remained almost at 100% during long-term cycling. The initial large capacity decay revealed the poor electrochemical stability of MoO_3_ electrode, which was due to the huge volume expansion and/or the structural change during the conversion reaction [91]. The rate capability displayed in Figure 7d for MOA showed significant capacity fading upon increasing the loading current. Moreover, after returning to the initial low C-rate (0.1C), the capacity did not return to its initial value and lost more than half of its value.

**Figure 8:**
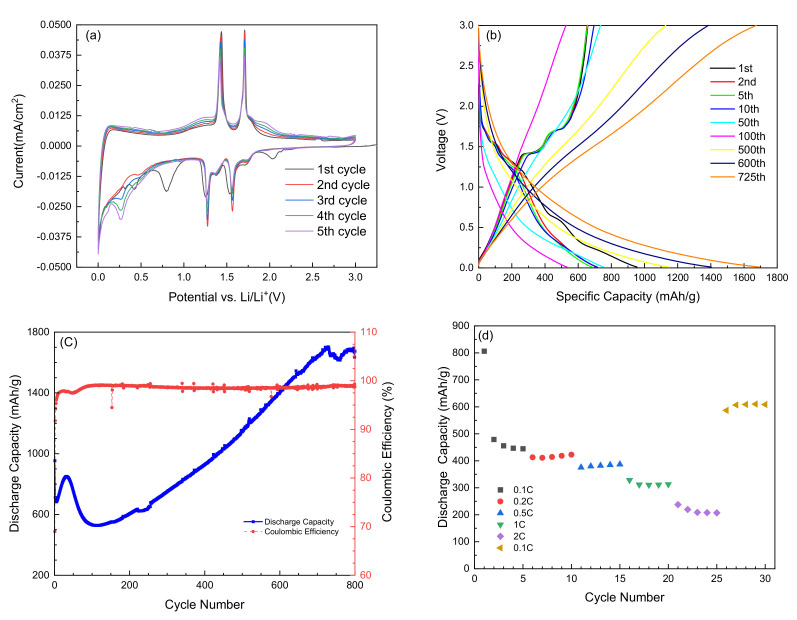
Electrochemical performance of MOV as anode material. (**a**) First five cyclic voltammograms recorded at scanning rate of 0.01 mV s^−1^. (**b**) Galvanostatic charge/discharge curves of MoO_2_ performed at 1C-rate (~0.86 A g^−1^). (**c**) Specific discharge capacity and Coulombic efficiency as a function of cycle number performed at 1C-rate. (**d**) Rate capability.

Figure 8a shows the first five cyclic voltammograms of the MOV electrode material, which exhibited five cathodic peaks located at 2.03, 1.53, 1.25, 0.78, and 0.42 V vs. Li^+^/Li. Note that the cathodic peaks located at 2.03 and 0.78 V disappeared starting from the 2nd cycle, and it may be related to the reduction in solution species and formation of a solid electrolyte interphase (SEI) on the anode surface, while the one at 0.42 V shifted to lower potential. The two strong cathodic peaks at 1.25 and 1.53 V were maintained in the subsequent cycles with a slight shift toward higher potentials (1.27 and 1.56 V, respectively). This high potential shift makes them close to the two strong anodic peaks at 1.43 and 1.7 V. A decrease in the potential difference Δ*E* between the redox peaks started from the 2nd cycle to 0.16 and 0.14 V instead of 0.18 and 0.17 observed in the 1st cycle. In the subsequent cycles, these voltage sets 1.27/1.43 and 1.56/1.7 V were assigned to the reversible phase transitions (monoclinic–orthorhombic–monoclinic) of partially lithiated Li_x_MoO_2_ during Li intercalation (discharge) and de-intercalation (charge) processes, which are in a good agreement with previous reports [92,93,94,95].

It is worth noting also that the CV curves starting from the 2nd cycle to the 5th cycle almost overlapped. This demonstrates that the as-prepared material (i.e., the mixture of MoO_2_ and Mo_n_O_3n-1_ suboxides) had good stability and reversibility for lithium-ion insertion and extraction during the first several cycles. Figure 8b illustrates the galvanostatic discharge–charge profiles of the MOV anode material tested at 1C-rate (~0.86 A g^−1^) in a potential window 0.01–3.0 V vs. Li^+^/Li. Two prominent plateaus at 1.25 and 1.53 V were observed during discharge process besides other small plateaus. The first lithiation mechanism was an insertion-type reaction that took place in the bulk and amorphous MoO_2_ electrodes with only one-electron reduction as described (see Equation (3)) [46]. The second step was a conversion reaction that gradually resolved Li_x_MoO_2_ as described by Equation (4) [47], showing the formation of metallic Mo and Li_2_O. In the charge curve, two plateaus were evidenced at 1.43 and 1.70 V and assigned to the deintercalation of Li^+^ from Li_x_MoO_2_ framework. The disappearance of small plateaus starting from the 2nd cycle is related to an irreversible structural change suggesting that part of Li^+^ cannot be extracted during the charge process [69]. Starting from the 2nd cycle, discharge and charge redox plateaus were clearly observed as noticed in the cyclic voltammograms. A voltage upgrading was detected upon cycling the MoO_2_ phase, which did not exist for MoO_3_. Figure 8c exhibits the electrochemical performance of the MOV anode material cycled at 1C-rate. An initial discharge capacity around 900 mAh g^−1^ was delivered in the 1st cycle, which decreased to ~500 mAh g^−1^ after 100 cycles and then increased on subsequent cycles reaching a value of 1625 mAh g^−1^ after 700 cycles. The Coulombic efficiency increased also from 93% to almost 99.4% after 100 cycles This better electrochemical behavior of the MOV electrode in comparison with that of MOA is attributed to several factors: (i) the smaller size of the nanoparticles, (ii) the presence of highly conductive suboxide phases, (iii) the higher BET specific surface area, (iv) the high intrinsic electrical conductivity of the MoO_2_ phase, and (v) the meso-porosity. The importance of cycling at a high C-rate was also evidenced in Figure 8d, showing the rate capability of the MOV electrode. The rate capability of the MOV electrode is shown in Figure 8d. When the C-rate increased from 0.1C to 2C, the discharge capacity decreased from approximately 450 mAh g^−1^ to approximately 200 mAh g^−1^. However, when the rate returned back to 0.1C after 2C testing, the capacity also returned back to approximately 600 mAh g^−1^, which is slightly higher than the capacity during the initial 0.1C test. This slight increase was consistent with the capacity change pattern shown in Figure 8c. The capacity slightly increased from the 5th cycle to about the 50th cycle.

The gradual increase in the discharge capacity and additional capacities beyond the correlating theoretical value upon long-term cycling is worthy to be discussed. This characteristic is common in a large number of conversion reaction metal-oxide anode materials [96,97,98,99,100,101,102,103,104,105,106]. Keppeler and Srinivasan stated that the mechanism leading an experimental capacity larger than the theoretical value remains speculative [98]. The literature reveals a lithium storage capacity higher by 10–100% at high current densities of 30–2000 mA g^−^^1^ after being tested beyond 50 cycles [96,97]. Different capacity shapes have been reported that exhibit additional capacity occurrences. Cobalt oxides frequently show a type I (mount-shape) capacity profile [99], type II (upward-shape) is observed for additional capacity occurrence for Fe- or Mn-oxide-based electrodes [100], type III (U-shape) is a typical capacity profile found for several cases when the anode material contains Mn or Fe [101], and iron-oxide-based electrodes tend to form a type IV (horizontal-shape) capacity profile [96]. Here, the MOV negative electrode material exhibited a capacity profile type III with a pronounced U-shape. The specific discharge capacity was almost twice the theoretical value after 800 cycles monitored at a high 1C-rate with a Coulombic efficiency, which remained constant at 99.4%. This is in contrast with the type IV profile reported by Shi et al. [102] for the mesoporous MoO_2_ electrode synthesized at 500 °C via a nanocasting strategy. However, the self-assembled porous MoO_2_/graphene microspheres, fabricated by Palanisamy et al. [103], exhibited a weak U-profile when cycled at a low current rate (C/10). A different capacity profile (upward-shape) was reported by Tang et al. [104] for an MoO_2_–graphene nanocomposite electrode cycled at 100 mA g^−1^. Thus, not only the morphology plays an important role in the excess capacity but also the operating mode is modifying the electrochemical performance upon long-term cycling of nanostructured oxides.

After the 100th test, the MOV anode displayed a gradual increase in specific capacity during cycling (Figure 8c). This anomalous behavior can be attributed to: (i) the activation of the porous structure with nano-cavities; the presence of numerous mesopores might be beneficial for the gradual access of the electrolytes in the porous structure of the electrode, and (ii) an additional Li-ion accommodation through reactions with the grain boundary phase in nanostructures; other scenarios associated with additional capacities, such as electrode/electrolyte interphases and electrocatalytic effect of metallic particles, have been identified [98]. The existence of numerous mesopores might be beneficial for more electrolytes accessing in the porous framework of the electrode, which favors the Li^+^ insertion/extraction process. Such a characteristic was evidenced in cobalt-based anodes [105], MnO/graphene composite [100], and graphene-wrapped Fe_3_O_4_ [106]. In MnO_x_ anodes, it might be based on mixed effects such as transition-metal cluster aggregation and formation of defects and deformation [101].

Table 5 summarizes the electrochemical performance of various MoO_2_ anode materials prepared by various synthetic processes [107,108,109,110,111,112,113,114,115,116,117,118,119,120,121,122,123,124,125]. The different strategies demonstrate the ability to mitigate the particle pulverization as a consequence of Li insertion/extraction and improve the MoO_2_ electrochemical performance via the fabrication of nanocomposites including carbonaceous materials. The particle size reduction results in the transport path shortening for both ions and electrons, while the carbonaceous matrix maintains high conductivity, large surface area, and chemical stability. The MoO_2_-based composites studied as lithium battery anodes involve various forms including mesoporous and monolith MoO_2_; nanostructured powders such as nanowires (NWs), nanospheres (NSs), hollow spheres (HSs), and nanobelts (NBs); MoO_2_/carbon materials; various binary composites. Thermo-electrochemical activation of MoO_2_ is also an attractive synthetic approach. A comparison of the electrochemical properties of these anode materials shows that the MoO_2_/Mo_n_O_3n–1_ composite prepared by a simple sol-gel technique assisted by a green chelator exhibits the best performance.

**Figure 9:**
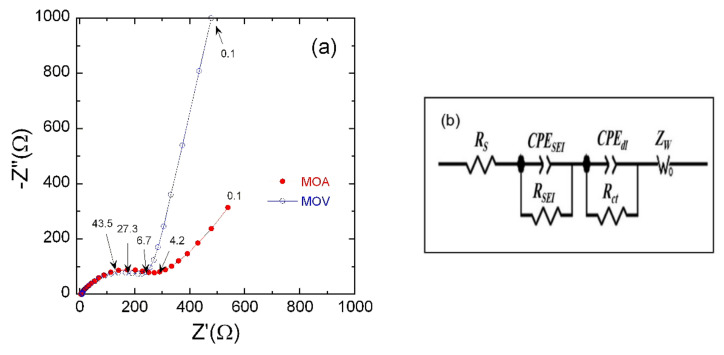
(**a**) Nyquist plots of fresh cells including the MOA and MOV electrodes. (**b**) The equivalent circuit model used for EIS data fitting.

To further investigate the electrochemical kinetics as well as characterize the improved electrochemical properties of MOA and MOV negative electrode materials, EIS measurements were carried out using a fresh cell. Figure 9a shows the Nyquist plots for the MOA and MOV electrodes. The equivalent circuit model (Figure 9b) used to analyze the EIS results is composed of a series of four elements: the cell resistance *R*_s_, a resistance in parallel with a constant phase element corresponding to the solid electrolyte interphase (*SEI*) layer, a second *R*-*CPE* parallel component, which figures out the charge transfer process, and finally the diffusion Warburg component (*Z*_W_). All Nyquist plots can be decomposed as follows: (i) the intercept at high frequency with the *Z*’-axis is related to the uncompensated ohmic resistance of the cell (*R*_s_); (ii) in the high-frequency region, the first depressed semicircle is associated with the SEI (*R*_SEI_, *CPE*_SEI_); (iii) a second depressed semicircle in the medium-frequency region is ascribed to the charge transfer impedance and the interfacial capacitance at the electrode/electrolyte interface (*R*_ct_, *CPE*_dl_); finally, (iv) in the low-frequency range, the inclined line is ascribed to the Li^+^-ion diffusion-controlled process characterized by the Warburg impedance. The values of *R*_s_ for the two samples are quite small (~7 Ω) implying a negligible ohmic polarization of the MOA and MOV electrodes. The *R*_ct_ value is lower in the MOV material (211 Ω) compared to the MOA (272 Ω) electrode. This matches well with the electrochemical performance of MOV mentioned above. This is attributed to the presence of Mo suboxides in MOV electrode (mixture of MoO_2_, o-Mo_4_O_11_, m-Mo_8_O_23_, and o-Mo_9_O_26_), which leads to a significant increase in the electronic conductivity as compared to MOA.

**Figure 10:**
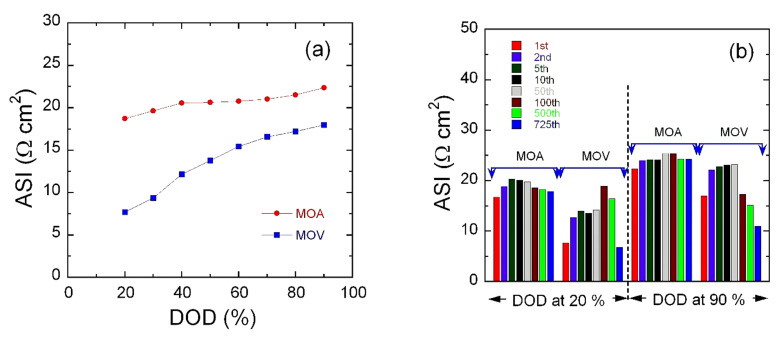
Area specific impedance (ASI) of the MOA and MOV as a function of depth of discharge (DOD) for the 1st discharge (**a**) and as a function of cycling for the MOA and MOV electrodes at 20% and 90% DOD (**b**).

More information on the change in the overall cell potential as a function of the depth-of-charge (DOD) can be obtained by evaluating the area-specific impedance (ASI expressed in Ω cm^2^) given by the relation [126,127]:

(7) $$ASI=A\frac{OCV-V_{cell}}{I},$$

where *A* is the cross-sectional area of the electrode, $\Delta V=OCV-V_{cell}$ is the potential change during current interruption for 60 s at each DOD step, and *I* is the current passing throughout the cell. ASI is affected also by ohmic drop, Li-ion diffusion through the electrolyte and solid-state diffusion within the electrode. This is like EIS measurements without the need to reach the equilibrium. Moreover, ASI could be more representative than data from EIS in terms of evaluation of the total cell resistance. However, ASI results confirmed the features observed by EIS. Figure 10a displays the variation of ASI for the MOA and MOV electrodes for the 1st discharge at 1C-rate. ASI values at 90% DOD are 22 and 16Ω cm^2^ for the MOA and MOV samples, respectively. The curves in Figure 10a indicate that, during battery discharging, the charge–transfer resistance is dependent on DOD. To further verify the effect of ASI on the electrochemical properties of MOA and MOV electrodes, ASI was calculated at various discharge cycles at 20% and 90% DOD as shown in Figure 10b. These results show that there were two different behaviors represented by an increase in the ASI values during the first five cycles, followed by a continuous decrease until the 725th cycle. The degree of decay in ASI values was much larger for MOV than for MOA at 20% DOD. By going to a deep discharge of 90% DOD, the situation looks rather different. ASI values increased after the 1st cycle then almost stabilizes until the 725th cycle for the MOA electrode. On the contrary, MOV showed smaller ASI values than MOA with a continuous reduction upon cycling upon shallow discharge (20% DOD). These results show that the lower ASI value was obtained for the MOV sample, and it is beneficial for the long-life cycling behavior.

## 4. Conclusions

This research article sheds light on the promising design strategies of molybdenum oxides for high kinetic energy storage. The green and facile preparation of nanosized molybdenum oxides (i.e., MoO_3_ and MoO_2_) by thermal decomposition of ammonium molybdate tetra hydrate (i.e., (NH_4_)_6_Mo_7_O_24_·4H_2_O) in air and in an inert atmosphere, respectively, has been demonstrated. The efficiency of the synthetic method is attributed to the use of orange peel extract as a chelator. The as-prepared MOA and MOV materials have the structure of MoO_3_ and MoO_2_+Mo_n_O_3n–1_ suboxides as estimated from XRD, XPS, and Raman spectroscopy. Thermal analysis emphasized the thermal stable phase of MoO_3_ up to approximately 800 °C and the presence of oxygen vacancies in the MOV sample. BET measurements show the mesoporous texture of molybdenum oxides; MOA had a lower specific surface area than MOV due to the easy crystal growth in the MoO_3_ phase. Electrochemical characterizations showed the outstanding properties in terms of capacity upgrading upon cycling for the MOV negative electrode material, which shows a pronounced U-shape capacity profile when cycled 800 times at 1C-rate. Finally, EIS and ASI experiments confirmed the superiority of the MOV (mixture of MoO_2_, Mo_4_O_11_, Mo_8_O_23_, and Mo_9_O_26_ phases) over the MOA (stoichiometric MoO_3_ insulator) sample as an anode material for Li-ion batteries.

## Figures and Tables

**Table 1:** Results of the Rietveld refinements for the MOA and MOV samples.

Crystal data	MOA	MOV
*Lattice parameters*		
*a (*Å*)*	3.69(5)	5.61(3)
*b (*Å*)*	13.84(8)	4.85(3)
*c (*Å*)*	3.95(9)	5.62(1)
*V (*Å*^3^)*	202.6	131.3
*L_c_* (nm)	29.5	45.6
*ε*× 10^−2^ (rd)	11.9	7.9
*Reliability factors*		
*R_p_ (%)*	10.9	8.1
*R_wp_ (%)*	16	11.2
*R_exp_*	9.1	7.7
*χ^2^*	3.1	2.1
*Materials fraction (mol%)*		
MoO_3_	100	0
MoO_2_	0	90.2
Mo_4_O_11_	0	2.2
Mo_8_O_23_	0	4.5
Mo_9_O_26_	0	3.1

**Table 2:** Reported Raman peak frequencies (cm^−1^) of M–O oxides.

α-MoO_3_		m-MoO_2_		o-Mo_4_O_11_		m-Mo_8_O_23_		o-Mo_9_O_26_	
Exp.	[81]	Exp.	[82]	Exp.	[82]	Exp.	[82]	Exp.	[82]
197 216 245 283 290 336 364 378 471 665 818 995	- 217 245 284 291 338 365 379 472 666 820 996	126 203 228 346 362 458 470 495 570 585 741	- 208 232 353 370 448 473 501 572 590 748	208 250 - 325 399 417 425 695 782 806 912 -	208 253 281 339 380 413 435 714 787 837 916 963	208 - 374 - - 656 - 912 950	208 222 373 384 592 654 875 918 951	208 - 544 - - - 782 912 - 950 -	208 465 575 622 637 679 761 906 931 951 989

**Table 3:** XPS analysis of the MOA and MOV samples.

Sample	Binding Energy (eV)										Average Mo Valence State
	Mo3p_3/2_	Mo3p_1/2_	O1s	Mo3d_5/2_				Mo3d_3/2_			
				Mo^4+^	Mo^5+^	Mo^6+^	Mo^4+^		Mo^5+^	Mo^6+^	
MOA	398.9	415.5	530.5	-	-	232.6	-		-	235.7	6.00
MOV	398.9	415.5	530.6	233.1	230.0	231.6	236.3		233.3	234.7	4.39

**Table 4:** BET specific surface area (*S*_BET_) and average pore size and pore volume of the MOA and MOV samples.

Sample	*S*_BET_(m^2^ g^−1^)	Pore Size(nm)	Pore Volume(cm^3^ g^−1^)	*L*_BET_(nm)
MOA	0.23	10	0.0012	3500
MOV	4.00	~2	0.0002	231

**Table 5:** Electrochemical performance of various MoO_2_ composites as anode materials for LIBs. The cycle number at which the specific capacity is reported is given in parenthesis.

Material	Synthesis	Reversible	Current	Reference
		Capacity	Rate	
		(mAh g^−1^)	(mA g^−1^)	
Nano MoO_2_	rheology	402	100 (40)	[118]
MoO_2_/Mo_2_N	reduction of MoO_3_	815	100 (150)	[119]
MoO_2_/graphene	chemical vapor deposition	986	50 (150)	[120]
MoO_2_/C	ion exchange	574	100 (100)	[121]
MoO_2_/C	carbothermal reduction	500	100 (50)	[108]
MoO_2_/C hollow spheres	solvothermal	580	200 (200)	[122]
Mesoporous MoO_2_	template casting	750	42 (30)	[103]
Activated MoO_2_	thermoelectrochemical activation	850	100 (30)	[123]
MoO_2_ HCSMSs	hydrolysis	420	50 (30)	[124]
W-doped MoO_2_	nanocasting	670	75 (20)	[111]
C/WO_x_/MoO_2_	hydrothermal	670	90 (50)	[125]
MoO_2_/C NWs	solvothermal	500	200 (20)	[109]
C/MoO_2_ NSs	hydrothermal+annealing	675	838 (30)	[113]
MoS_2_/MoO_2_	sulfur assisted	654	500 (80)	[115]
C/MoO_2_ NBs	hydrothermal+annealing	617	100 (30)	[110]
MoO_2_ monolith	morphosynthesis	719	200 (20)	[112]
α-MoO_3_@β-MnO_2_	two-step hydrothermal	286	6C (50)	[107]
MoO_2_/N-doped C NWs	calcination	700	2000 (400)	[114]
C-coated MoO_2_	hydrothermal	312	10000 (268)	[116]
MoO_2_/flexible C	electrospinning	451	2000 (500)	[117]
MoO_2_/Mo_n_O_3n-1_	sol-gel with green chelator	1600	800 (800)	this work

## Data Availability

Data are contained within the article.
